# A Novel Mechanism for Nitrosative Stress Tolerance Dependent on GTP Cyclohydrolase II Activity Involved in Riboflavin Synthesis of Yeast

**DOI:** 10.1038/s41598-020-62890-3

**Published:** 2020-04-07

**Authors:** Khairul Anam, Ryo Nasuno, Hiroshi Takagi

**Affiliations:** 1 Division of Biological Science, Graduate School of Science and Technology, Nara Institute of Science and Technology, 8916-5, Takayama-cho, Ikoma, Nara, 630-0192 Japan; 2 Research Center for Biotechnology, Indonesian Institute of Sciences, Jl. Raya Bogor KM 46, Cibinong, 16911 Bogor, West Java Indonesia

**Keywords:** Fungal physiology, Cell growth

## Abstract

The biological functions of nitric oxide (NO) depend on its concentration, and excessive levels of NO induce various harmful situations known as nitrosative stress. Therefore, organisms possess many kinds of strategies to regulate the intracellular NO concentration and/or to detoxify excess NO. Here, we used genetic screening to identify a novel nitrosative stress tolerance gene, *RIB*1, encoding GTP cyclohydrolase II (GTPCH2), which catalyzes the first step in riboflavin biosynthesis. Our further analyses demonstrated that the GTPCH2 enzymatic activity of Rib1 is essential for *RIB1*-dependent nitrosative stress tolerance, but that riboflavin itself is not required for this tolerance. Furthermore, the reaction mixture of a recombinant purified Rib1 was shown to quench NO or its derivatives, even though formate or pyrophosphate, which are byproducts of the Rib1 reaction, did not, suggesting that the reaction product of Rib1, 2,5-diamino-6-(5-phospo-d-ribosylamino)-pyrimidin-4(3 H)-one (DARP), scavenges NO or its derivatives. Finally, it was revealed that 2,4,5-triamino-1H-pyrimidin-6-one, which is identical to a pyrimidine moiety of DARP, scavenged NO or its derivatives, suggesting that DARP reacts with N_2_O_3_ generated *via* its pyrimidine moiety.

## Introduction

Nitric oxide (NO) is a small signaling molecule that plays various roles in a number of biological processes^1,2^. In mammalian cells, NO is generally produced by three different isoforms of NO synthase (NOS) from l-arginine and NADPH^3^. NOS consists of the oxygenase domain responsible for oxidation of l-arginine and the reductase domain which transfers electrons from NADPH to the oxygenase domain^4^. Some Gram-positive bacteria have enzymes called bacterial NOS (bNOS), which is homologous to the oxygenase domain of NOS, and which exerts its NOS activity *via* interaction with uncertain reductase proteins^5^. On the other hand, nitrate and nitrite serve as NO sources in plants, in which nitrate and nitrite are reduced to NO by nitrate reductase and nitrite reductase, respectively^6^. Nitrite is also reduced to NO by heme-containing proteins including hemoglobin or molybdopterin proteins such as xanthine oxidase *via* their nitrite reductase activity^7,8^. Furthermore, reduction of nitrite to NO is also catalyzed by the mitochondrial respiratory activity complex III and/or IV^9^.

NO activates soluble guanylyl cyclase (sGC) by binding to the heme in sGC. Activated sGC releases cyclic GMP, which is a second messenger leading to the relaxation of smooth muscle cells in mammals^10^. Additionally, NO induces the posttranslational modification of proteins, such as *S*-nitrosation. *S*-nitrosation is formed *via* the reaction of NO with thiyl radical or between NO^+^, which is one electron oxidized form of NO, and the thiol group of cysteine residues in the target protein^11^. Thiol group also undergoes *S*-nitrosation *via* transnitrosation, in which NO^+^ group is transferred from a sulfur atom in nitrosothiol compound to that in a target thiol compound. The NO-responsive transcription factor OxyR is activated *via* its *S*-nitrosation in *Escherichia coli*^12^. Furthermore, other reactive nitrogen species (RNS) including peroxynitrite, which is generated through the reaction of NO with superoxide anion^11^, nitrates tyrosine and/or tryptophan residues in proteins, thereby altering the protein functions^13^.

The biological functions of NO depend on its concentration. At a low or homeostatic level, NO is involved in physiological phenomena. For example, an appropriate level of NO in human cells promotes tolerance to oxidative stress, vasodilation of smooth muscle, and neurotransmitter release^14^. A low level of NO also plays a role in oxidative stress tolerance in bacteria^15^. In the budding yeast *Saccharomyces cerevisiae*, we found that NO confers high temperature stress tolerance through the activation of the copper-related transcription factor Mac1^16–18^. On the other hand, an excessive level of RNS including NO could cause nitrosative stress leading to cellular damage or death. NO generated under the highly oxidative condition induced by hydrogen peroxide treatment leads to apoptosis-like cell death in *S. cerevisiae*^19,20^. In terms of health and disease, nitrosative stress has been reported to be involved in various pathophysiological phenomena through damage of DNA, proteins, and lipids^2^.

Therefore, it is important for living organisms to control the intracellular and/or intercellular concentration of NO *via* the appropriate regulation of synthesis and degradation. A flavohemoglobin (fHb), which possesses FAD and heme, oxidizes or reduces NO to NO_3_^−^ or N_2_O, under aerobic or anaerobic conditions, respectively^21^. An *S*-nitrosoglutathione reductase (GSNOR) reduces *S*-nitrosylated cysteine residues on proteins to free cysteine residues and ammonium with the aid of thioredoxin (Trx) and thioredoxin reductase (Trr). fHb and GSNOR are widely conserved among microorganisms regardless of the presence of NOS orthologous genes, suggesting the importance of the defense system against excess levels of NO.

RNS, including NO and peroxynitrite, also function as anti-microbial agents against pathogenic bacteria, fungi, and yeasts. During infection, macrophages produce NO and superoxide by inducible NOS and NADPH oxidase, respectively, and then these two molecules react with each other to generate peroxynitrite with higher reactivity and the ability to kill pathogens. It has been reported that NO upregulates fHb expression in *Candida albicans* and that fHb is essential for infection of this pathogenic yeast to host cells by weakening the toxicity of NO released from the host^22–25^. Therefore, it is essential for infection to avoid nitrosative stress effectively through various strategies. In addition to fHb and GSNOR, nitrosothionein (NT), which is a small protein containing many cysteine residues, was recently identified as an NO scavenging molecule in the model fungus *Aspergillus nidulans*. NT traps NO with its cysteine residues and *S*-nitrosylated residues of NT are regenerated through the reduction system by Trx/Trr. The discovery of NT suggests the presence of further anti-nitrosative stress systems in various microbes, such as pathogenic and model bacteria, fungi, and yeasts, including *S. cerevisiae*.

Here, we screened and identified a novel nitrosative stress tolerance gene *RIB1*, which has been annotated as a gene encoding GTP cyclohydrolase II (GTPCH2) involved in riboflavin (RF) biosynthesis. Our further analyses suggested that the enzymatic reaction product of GTPCH2 scavenged RNS, which confers nitrosative stress tolerance on yeast cells.

## Results

### Identification of a novel nitrosative stress tolerance gene *RIB1*

**Figure 1:**
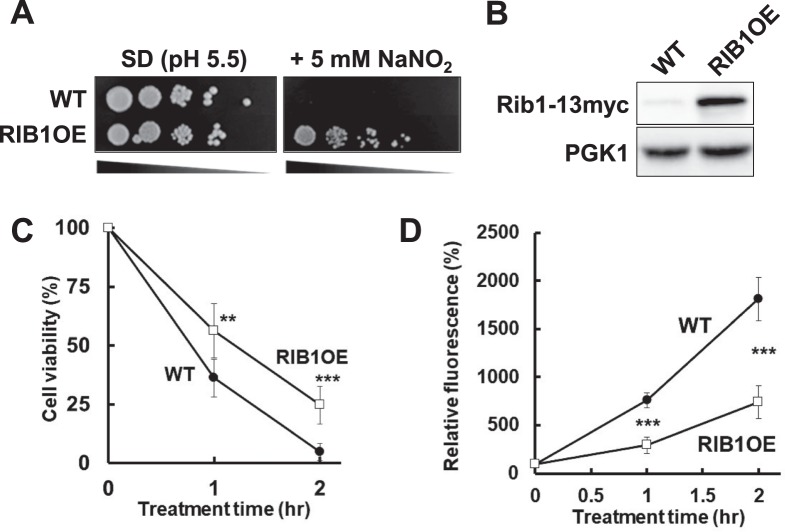
Overexpression of *RIB1* confers nitrosative stress tolerance on yeast cells. (**A**) Spot assay for nitrosative stress tolerance. Yeast cells grown until the exponential growth phase were serially diluted and then spotted onto acidified medium with NaNO_2_. (**B**) Confirmation of *RIB1* overexpression with Western blotting. (**C**) Yeast cells grown until the exponential growth phase were exposed to an NO donor NOC-5 (0.5 mM) and then plated on YPD medium, followed by counting the number of colonies to calculate cell viability. (**D**) Yeast cells grown until the exponential growth phase were treated with DAF-FM DA, followed by exposure to 0.5 mM NOC-5. The intracellular fluorescence intensity was analyzed by FCM. The values in panels C and D are the means and standard deviations of three independent experiments. ***p < 0.001; **p < 0.01 by Student’s *t* test.

In order to isolate genes involved in nitrosative stress tolerance, the screening was performed in acidified medium (pH 5.5) containing NaNO_2_, as previous studies reported^26,27^. It was previously shown that nitrite induces *S*-nitrosylation of proteins under acidic condition^28^. The growth rate of more than 160,000 colonies of transformants harboring the multi-copy plasmid library, which contained the *Sau*3AI-digested genomic DNA of the *S. cerevisiae* X2180-1A strain, on medium containing NaNO_2_ as an RNS donor was evaluated, and approximately 400 colonies of transformants were selected. The isolated transformants were further analyzed by a spotting assay, and their plasmid dependence was confirmed (Fig. S1A). DNA sequencing analysis indicated that the plasmids extracted from all four of the finally selected candidate clones contained the *RIB1* gene, which has been reported to be involved in RF biosynthesis^29^ (Fig. S1B). To confirm the involvement of *RIB1* in nitrosative stress tolerance, the growth rate of *RIB1*-overexpressing (RIB1OE) strains on acidified medium containing NaNO_2_ was examined (Figs. 1A,B, and S3). As a result, the RIB1OE strain, which expressed a high level of the Rib1 protein, showed faster growth than the wild-type (WT) strain, indicating that the overexpression of *RIB1* enhances the nitrosative stress tolerance of yeast cells.

Subsequently, RIB1OE cells were treated with an NO donor, 3-[2-hydroxy-1-(1-methylethyl)-2-nitrosohydrazinyl]-1-propanamine (NOC-5), in a liquid SD medium, and then cell viability was measured. As shown in Fig. 1C, relative cell viability was significantly higher in RIBOE cells than in WT cells after treatment with NOC-5. Additionally, the intracellular NO level after treatment with NOC-5 was estimated by flow cytometry using cells stained with an NO specific fluorescence probe 4-amino-5-methylamino-2′,7′-difluorofluorescein diacetate (DAF-FM DA) (Fig. 1D). The results showed that the intracellular fluorescence increased with time. Importantly, the intracellular fluorescence of the RIB1OE strain increased significantly more slowly than that of the WT strain. These results suggest that the overexpression of *RIB1* confers nitrosative stress tolerance on yeast cells, by reducing the intracellular level of RNS including NO.

### Riboflavin is not involved in *RIB1*-dependent nitrosative stress tolerance

**Figure 2:**
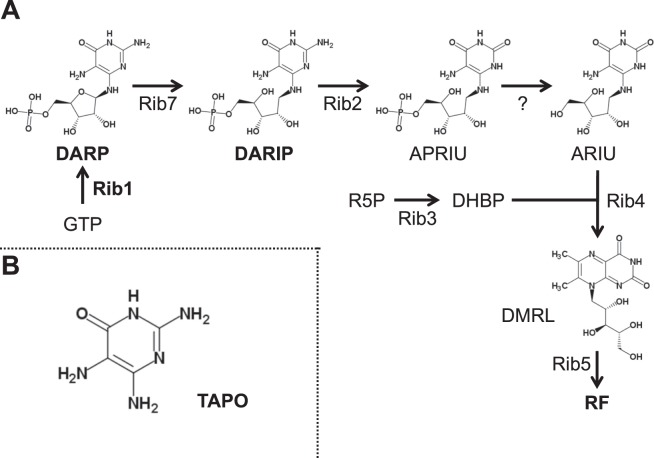
The RF biosynthesis pathway and the chemical structure of related compounds. (**A**) The RF biosynthesis pathway in *S. cerevisiae*. Protein names: Rib1, GTP cyclohydrolase II; Rib2, bifunctional DRAP deaminase; Rib3, 3,4-dihydroxy-2-butanone-4-phosphate synthase; Rib4, lumazine synthase; Rib5, riboflavin synthase; Rib7, 2,5-diamino-6-(ribosylamino)-4(3 H)-pyrimidinone 5′-phosphate reductase. Metabolite names: DARP, 2,5-diamino-6-(5-phospho-d-ribosylamino)pyrimidin-4(3 H)-one; DARIP, 2,5-diamino-6-(5-phospho-d-ribitylamino)pyrimidin-4(3 H)-one; APRIU, 5-amino-6-(5′-phospho-d-ribitylamino)uracil; ARIU, 5-amino-6-(1-d-ribitylamino)uracil; R5P, D-ribulose 5-phosphate; DHBP, L-3,4-dihydroxybutan-2-one 4-phosphate; DMRL, 6,7-dimethyl-8-(d-ribityl)lumazine. The chemical structure of (**B**) TAPO.

The *RIB1* gene encodes GTPCH2, which catalyzes the first step in the RF biosynthesis pathway^29,30^ (Fig. 2A). In this pathway, GTPCH2 converts GTP into 2,5-diamino-6-(5-phospo-d-ribosylamino)-pyrimidin-4(3 H)-one (DARP) and then DARP is further modified by several enzymes to generate the final product, RF. Though RF is a precursor for FAD, which is a cofactor of a widely conserved NO dioxygenase fHb, there have been no reports showing the involvement of GTPCH2 and/or RF biosynthesis in nitrosative stress tolerance^29,31^. Therefore, we investigated whether RF is required for *RIB1*-dependent nitrosative stress tolerance.

**Figure 3:**
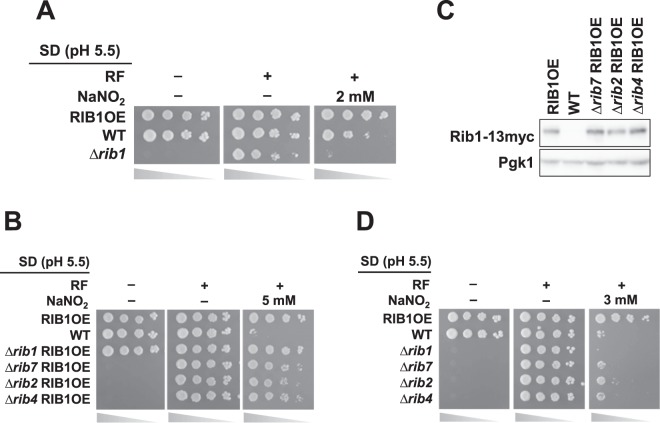
Relationship of the RF biosynthesis with *RIB1*-dependent nitrosative stress tolerance. Effect of (**A**) RF supplementation or (**B**) the *RIB* gene disruption on *RIB1*-dependent nitrosative stress tolerance. (**A,B**) Yeast cells grown under the exponential growth phase were serially diluted and spotted onto acidified NaNO_2_ medium in the presence or absence of RF. (**C**) Confirmation of *RIB1* overexpression in each *RIB* gene-deleted strain by Western blotting. (**D**) Effect of the *RIB* gene disruption on nitrosative stress resistance, analyzed same as (**A,B**).

To determine whether RF promotes nitrosative stress tolerance, the growth rates of WT, RIB1OE, and ∆*rib1* strains were evaluated on acidified medium containing NaNO_2_ in the presence or absence of RF. As shown in Fig. 3A, the RF auxotroph ∆*rib1* strain did not grow on SD medium, but grew at a normal rate on the medium with RF, indicating the incorporation of RF into yeast cells under the condition tested here. Interestingly, ∆*rib1* cells showed significantly higher sensitivity to NaNO_2_ than WT cells even in the presence of RF. Additionally, the effect of *RIB1* overexpression on the *YHB1*-disrupted strain was examined (Fig. S2). The result showed that the growth on the acidified nitrite medium was improved by overexpression of *RIB1* even in the absence of *YHB1*. These results indicate that the *RIB1*-dependent nitrosative stress tolerance functions independently of RF and RF-dependent NO dioxygenase fHb.

To further analyze the mechanism underlying *RIB1*-dependent nitrosative stress tolerance, we examined the genetic interactions between *RIB1* and other *RIB* genes encoding enzymes in the RF synthesis pathway. The growth rate assay of RIB1OE and its variant lacking each *RIB* gene showed that the overexpression of *RIB1* enhances nitrosative stress tolerance regardless of the presence of other *RIB* genes, on the other hand, deletion of each *RIB* gene did not alter the expression level of *RIB1* significantly (Figs. 3B,C, and S3). Additionally, the nitrosative stress tolerance of each *RIB* gene disruptant was evaluated on the acidified NaNO_2_ medium in the presence of RF (Fig. 3D). The result showed that the deletion of *RIB1* caused higher sensitivity to nitrosative stress, although the *RIB7*, *RIB2*, and *RIB4* disruptants showed the same phenotype as did WT. These results indicate that, among the genes encoding RF synthesis enzymes, *RIB1* is an only or major gene which confers nitrosative stress tolerance on yeast cells without any cooperation from and/or interaction with other *RIB* genes or the activities of enzymes in the RF synthesis pathway.

### GTP cyclohydrolase II activity is required for *RIB1*-dependent nitrosative stress tolerance

**Figure 4:**
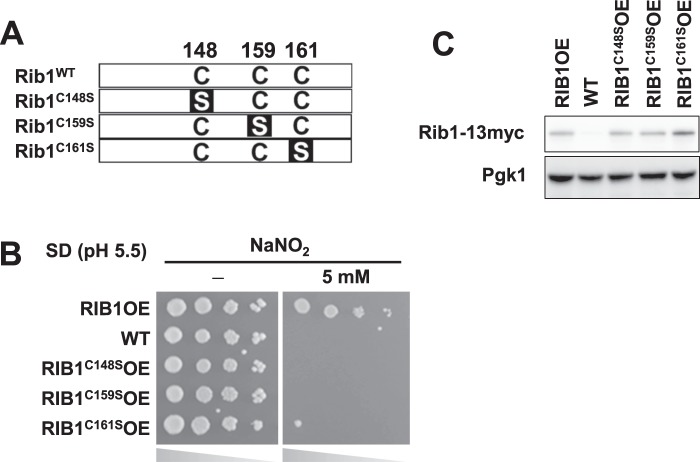
Importance of Rib1 enzymatic activity on *RIB1*-dependent nitrosative stress tolerance. (**A**) Position of conserved cysteine residues and the corresponding amino acid substitutions in Rib1. (**B**) Nitrosative stress tolerance of yeast cells expressing Rib1 mutants. Yeast cells grown until the exponential growth phase were serially diluted and spotted onto the acidified NaNO_2_ medium. (**C**) Confirmation of each Rib1 mutant by Western blotting.

The *RIB1* gene product, GTPCH2, has been reported to exert its activity using Zn^2+^ coordinated by three conserved cysteine residues^30^. Therefore, we analyzed the effects of amino acid substitution from cysteine to serine at positions 148, 159, or 161, which are highly conserved, on *RIB1*-dependent nitrosative stress tolerance (Fig. 4A). Our growth rate evaluation of each strain overexpressing Cys148Ser-, Cys159Ser-, or Cys161Ser-Rib1 on the acidified NaNO_2_ medium showed that none of the strains expressing mutant Rib1 conferred nitrosative stress tolerance, although the cells overexpressing WT Rib1 clearly enhanced nisrosative stress tolerance (Fig. 4B). On the other hand, these amino acid substitution did not decrease the expression level of *RIB1* (Figs. 4C and S3). These results indicate that Rib1 requires GTPCH2 activity to protect yeast cells from nitrosative stress.

### DARP, the reaction product by Rib1, scavenges RNS

**Figure 5:**
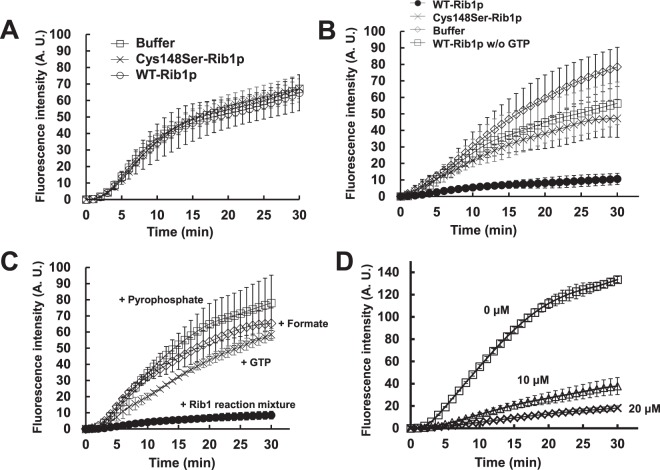
Analysis of NO scavenging activity of Rib1 and its reaction products. DAF-FM was reacted with an NO donor NOC-5 (0.5 mM) in the presence or absence of various additives. (**A**) Rib1 reaction mixture without GTP containing buffer control, Cys148Ser-, or WT-rRib1p was added. (**B**) The Rib1 reaction mixture with GTP containing buffer control, Cys148Ser-, or WT-rRib1p was added. The Rib1 reaction mixture without GTP containing WT-rRib1p was also added as a control. (**C**) Each of the reaction products by Rib1, or (**D**) various concentrations of TAPO was added. The fluorescence intensity was monitored over time. The values are the means and standard deviations of three independent experiments.

Our genetic analyses indicated that other *RIB* genes were not involved in *RIB1*-dependent nitrosative stress tolerance. This led us to hypothesize that the Rib1 enzyme and/or its enzymatic reaction products are involved in resistance against RNS including NO. To examine this hypothesis, we purified a recombinant Rib1 (rRib1p) enzyme using the *Escherichia coli* expression system. The purified rRib1p showed specific activity of 3.6 U/mg. First, we tested the NO degrading activity of rRib1p. An NO specific fluorescence probe 4-amino-5-methylamino-2′,7′-difluorofluorescein (DAF-FM) was reacted with NOC-5 in the presence or absence of WT- or mutant Cys148Ser-rRib1p and the fluorescence was monitored over time (Fig. 5A). The results showed that the fluorescence increased with time regardless of the presence of active WT-rRib1p, indicating that Rib1 does not degrade NO directly. Next, we examined the NO scavenging activity of the Rib1 enzymatic reaction products. DAF-FM was reacted with NOC-5 in the presence of the reaction mixture of rRib1p using GTP as a substrate, which was prepared as described in Methods section, and the fluorescence was monitored (Fig. 5B). Interestingly, the reaction mixture of WT-rRib1p dramatically inhibited the time-dependent increase of fluorescence induced by NOC-5. On the other hand, the reaction mixture of inactive mutant Cys148Ser-rRib1p or that of WT-rRib1p without GTP did not suppress the increase in fluorescence, same as the buffer control. These results suggested that the enzymatic reaction products of Rib1 scavenge NO or its derivatives. The GTPCH2 activity of Rib1 catalyzes the reaction using GTP and water as a substrate to generate its products, which are DARP, formate, and pyrophosphate. Therefore, the effect of each product of the Rib1 enzymatic reaction was examined by the same method as above (Fig. 5C). The time-dependent monitoring demonstrated that the reaction mixture of WT-rRib1p inhibited the fluorescence increase, whereas neither the Rib1 substrate nor each reaction product, including formate, pyrophosphate, and GTP inhibited it, suggesting that DARP is a scavenger of NO or its derivatives.

DARP consists of a pyrimidine derivative, ribose, and phosphate (Fig. 2A). Among them, ribose and phosphate also exist in the chemical structure of GTP, which has no activity to scavenge NO, raising the possibility that the pyrimidine moiety of DARP reacts with NO or its derivatives directly. Therefore, an NO scavenging activity of 2,4,5-triamino-1H-pyrimidin-6-one (TAPO), which is identical to the pyrimidine moiety of DARP, was analyzed (Fig. 2B). DAF-FM was reacted with NOC-5 in the presence of TAPO with varied concentrations and the fluorescence was monitored (Fig. 5D). These results showed that TAPO inhibited the increase of fluorescence in a dose-dependent manner, which let us conclude that DARP scavenges NO or its derivatives through its pyrimidine moiety to reduce the toxicity of nitrosative stress.

## Discussion

In this study, we demonstrated that the *RIB1* gene encoding GTPCH2, which is responsible for the first step in RF biosynthesis, confers nitrosative tolerance on the yeast *S. cerevisiae*. Our further analyses indicated that DARP, the reaction product of GTPCH2 activity, scavenges RNS *via* its pyrimidine derivative moiety (Fig. 5). There has been no report that DARP or other RF metabolism intermediates scavenge RNS or attenuate nitrosative stress. Thus, *RIB1*/DARP was here identified for the first time as a novel gene/metabolite that plays a role in inducing the nitrosative stress tolerance mechanism in *S. cerevisiae*.

From the results of the nitrosative stress tolerance analyses, Δ*rib1* cells showed higher sensitivity to the acidified NaNO_2_ condition than WT cells, indicating that the physiological expression level of *RIB1* contributes to nitrosative stress tolerance. This is in contrast with our previous microarray analysis^17^, which indicated that *RIB1* is not induced by NO treatment. On the other hand, fHb, one of the major NO detoxifying enzymes, is upregulated after exposure to NO through the activation of the transcription factor Fzf1 in *S. cerevisiae* or Cta4 in *Candida albicans*^31–33^. NT is also induced by treatment with NO in *A. nidulans*^27^. From these facts, we concluded that *RIB1*-dependent nitrosative stress tolerance functions as a basal cell protection system against RNS.

GTPCH2 is conserved among a wide variety of organisms including yeasts, fungi, bacteria, and plants, but not in human (Table 1). Extensive studies revealed that the disruption of RNS tolerance genes leads to a decrease in the infection efficiency of pathogenic microorganisms. Therefore, the *RIB1*-dependent RNS tolerance system could be a promising target for developing novel anti-fungal or bacterial drugs.

**Table 1:** The BLAST search of homologous proteins to Rib1 of *S. cerevisiae*.

Description	Organisms	Query Coverage	E value	Identity
Probable GTP cyclohydrolase-2	*Candida glabrata*	85%	5e–175	79.25%
GTP cyclohydrolase II	*Candida albicans*	84%	8e–128	65.20%
GTP cyclohydrolase II	*Aspergillus fumigatus*	79%	3e–102	55.82%
GTP cyclohydrolase II	*Schizosaccharomyces pombe*	81%	3e–101	55.12%
Hypothetical protein	*Aspergillus nidulans*	73%	1e–94	55.27%
Riboflavin biosynthesis protein	*Arabidopsis thaliana*	49%	5e–36	47.67%
GTP cyclohydrolase II	*Escherichia coli*	50%	6e–35	44.00%
Fused protein, GTP cyclohydrolase II	*Bacillus subtilis*	49%	3e–33	46.20%

Protein BLAST search (https://blast.ncbi.nlm.nih.gov/Blast.cgi) was performed using amino acid sequence of Rib1 from *S. cerevisiae* S288c strain as a query sequence.

The results of our NO quenching assay suggest that the TAPO moiety of DARP reacts with NO or its derivatives directly. TAPO is a pyrimidine derivative and has two amino groups at the ortho position on the pyrimidine ring. Its structure, containing two amino groups at the ortho position on the aromatic ring, is similar to those of several NO_x_-reactive molecules, such as NO probe diaminofluoresceine derivatives (DAFs) and 2, 3-diaminonaphthalene (DAN), which is used to measure nitrite. DAFs or DAN reacts with dinitrogen trioxide N_2_O_3_, which is generated by autoxidation of NO or from nitrous acid produced by protonation of nitrite, respectively, through their aromatic amino groups, and then the resultant triazole structure fluoresce^34,35^. These findings raise the hypothesis that DARP reacts with N_2_O_3_, which is generated under nitrosative stress conditions, *via* two amino groups in the TAPO moiety and form a triazole derivative, which is expected to be 8-azaguanine or its derivatives.

In the RF biosynthesis, one more compound harboring a same structure as TAPO is 2,5-diamino-6-(5-phospho-d-ribitylamino)pyrimidin-4(3 H)-one (DARIP), which is generated from DARP by the *RIB7*-encoded reductase (Fig. 2A). Our spot assay showed that the deletion of *RIB7* did not affect nitrosative stress tolerance in both the WT and *RIB1*-overexpressing strains (Fig. 3B,D). On the other hand, the *RIB1*-disrupted cells exhibited higher nitrosative sensitivity than WT cells (Fig. 3A,D). The RF synthesis pathway indicates that Δ*rib1* or Δ*rib7* cells are unable to synthesize DARP/DARIP or DARIP, respectively, whereas the previous study reported that the *RIB7* deficient mutant accumulated DARP^36^. It is possible that DARIP is decreased and DARP is alternatively increased in Δ*rib7* cells, leading to the same level of nitrosative stress tolerance of Δ*rib7* cells as WT cells. We did not measure the intracellular contents of DARP and DARIP in this work. Thus, it is suggested that not only DARP but also DARIP, which possess the TAPO moiety, function as RNS scavengers to attenuate nitrosative stress.

## Methods

### Strains, plasmids, and medium

The strains, primers, and plasmids used in this study were listed in Tables 2, S1, and Table 3, respectively. The yeast *S. cerevisiae* BY4741 strain (*MAT*a *his3*Δ*1 leu2*Δ0 *met15*Δ0 *ura3*Δ0) was used as a host strain to construct yeast strains used in this study. BY4741 strain was transformed with the DNA fragment amplified by PCR using the plasmid pFA6a-13myc-kanMX6 as a template and the primers listed in Table S1, to generate the strain expressing Rib1 fused with 13-myc-tag at its C-terminus from the genome, described as WT strain in this study. The DNA fragments amplified by PCR using the plasmid pFA6a-kanMX6 as a template and each corresponding primer listed in Table S1 were introduced into BY4741 to construct each of the *RIB* gene disruptants. *E. coli* DH5α or Rosetta (DE3) strain was used to construct plasmids or express rRib1p, respectively.

**Table 2:** Yeast strains used in this study.

Strains	Genotype	Introduced plasmids
BY4741	*MATa his3Δ1 leu2Δ0 met15Δ0 ura3Δ0*	pAG415GPD-ccdB
WT	BY4741 *RIB1–13myc::kanMX6*	pAG415GPD-ccdB
RIB1OE	WT	pAG415GPD-RIB1-13myc
Δ*rib1*	BY4741Δ*rib1::kanMX6*	pAG415GPD-ccdB
Δ*rib1* RIB1OE	BY4741Δ*rib1::kanMX6*	pAG415GPD-RIB1-13myc
Δ*rib2* RIB1OE	BY4741Δ*rib2::kanMX6*	pAG415GPD-RIB1-13myc
Δ*rib4* RIB1OE	BY4741Δ*rib4::kanMX6*	pAG415GPD-RIB1-13myc
Δ*rib7* RIB1OE	BY4741Δ*rib7::kanMX6*	pAG415GPD-RIB1-13myc
RIB1^C148S^OE	Rib1-myc	pAG415GPD-RIB1^C148S^-13myc
RIB1^C159S^OE	Rib1-myc	pAG415GPD-RIB1^C159S^-13myc
RIB1^C161S^OE	Rib1-myc	pAG415GPD-RIB1^C161S^-13myc

All yeast strains were transformed with pRS416-CgHIS3-MET15 to complement the nutrient anxotrophy, in addition to the plasmids listed above.

**Table 3:** List of plasmids used in this study.

Plasmids for *S. cerevisiae*	Marker	Type	Promoter	Expressed protein
pAG415GPD-RIB1-13myc	*LEU2*	*CEN*	*GPD*	Rib1-13myc
pAG415GPD-ccdB	*LEU2*	*CEN*	—	—
pRS416-CgHIS3-MET15	*URA3*, *HIS3*, *MET15*	*CEN*	—	—
**Plasmids for** ***E. coli***	**Marker**	**Description**		
pET53-RIB1	*Amp*^*R*^	Expression of His-tagged Rib1, induced by IPTG		

The *RIB1* coding region including the myc-tag amplified from the genome of WT strain was inserted to the cloning site on the plasmid pAG415GPD-ccdB by the BP and LR reactions of Gateway technology (Invitrogen) following the manufacturer’s protocol, to generate the plasmid pAG415GPD-RIB1-13myc, which was introduced to each host strain to overexpress *RIB1*. The plasmids pAG415GPD-RIB1^C148S^-13myc, pAG415GPD-RIB1^C159S^-13myc, and pAG415GPD-RIB1^C161S^-13myc were constructed as follows. The DNA fragments were amplified through PCR using the primers listed in Table S1 and pDONR221-RIB1-13myc, which was an intermediate during construction of pAG415GPD-RIB1-13myc, as a template. The generated PCR products were treated with *Dpn*I, phosphorylated with T4DNA kinase, and subjected to ligation reaction for self-ligation, followed by LR reaction with pAG415GPD-ccdB to construct each expression plasmid. The resultant plasmids were introduced into WT strain to overexpress each Rib1 mutant with amino acid substitution Cys148Ser, Cys159Ser, or Cys161Ser, respectively. The plasmid pRS416-CgHIS3-MET15, which expresses the *HIS3* gene of *Candida glabrata*, *URA3*, and *MET15*, was introduced to each yeast strain to complement its auxotrophy.

To express rRib1p in *E. coli* cells, pET53-RIB1 was constructed through the BP and LR reaction as described above from BG1805-RIB1, which was derived from Yeast ORF Collection (Open Biosystems). pDONR221-RIB1, an intermediate during construction of pET53-RIB1, was used as a template to construct pET53-RIB1^C148S^, which expresses mutant Rib1 with the amino acid substitution Cys148Ser, with the same methods as described above.

Yeast cells were cultivated in YPD (1% yeast extract, 2% peptone, and 2% glucose) or SD (0.17% yeast nitrogen base without amino acid and ammonium sulfate (Difco), 0.5% ammonium sulfate, and 2% glucose, pH 5.5) medium, with 200 mg/L of G418 if necessary. LB (0.5% yeast extract, 1% tryptone, and 1% NaCl) medium was used for *E. coli* cultivation, with or without 200 mg/L ampicillin, 100 mg/L kanamycin, and/or 35 mg/L chloramphenicol. To prepare solid medium, 2% agar was added to each medium.

### Screening of nitrosative stress tolerance genes

The plasmid libraries used in this study, which was constructed by ligation of *Sau*3AI-digested genomic DNA of *S. cerevisiae* X2180-1A strain into Yep51B vector, were obtained from National BioResource Project (NBRP)^36^. The library was introduced into BY4741 strain harboring pRS416-CgHIS3-MET15 and then spread onto SD medium (pH 5.5) containing 8 mM NaNO_2_. After 2–4 days culture at 30 °C, colonies growing faster were picked up. Isolated clones were subjected to spot assay on SD medium (pH 5.5) with 0 to 5 mM NaNO_2_ for more detail evaluation. Plasmids extracted from finally selected clones were introduced into BY4741 strain again to examine the plasmid dependency of their nitrosative stress tolerance. After confirmation of the plasmid dependency, each isolated plasmid library was sequenced to identify the inserted DNA region in it.

### Spot test

Yeast cells cultured in SD medium until early exponential growth phase were serially diluted and then spotted onto SD medium (pH 5.5) with or without NaNO_2_, in the presence of 133 μM RF if necessary.

### Cell viability assay

Yeast cells cultured in SD medium (pH 5.5) until early exponential phase were treated with 0.5 mM NOC-5 for 0, 1, or 2 h and then spread on YPD solid medium. After cultivation at 30 °C for 1 to 2 days, the number of generated colonies was counted. Cell viability was calculated as follows: (the number of colonies after incubation with NOC-5)/(the number of colonies after incubation without NOC-5) × 100.

### Measurement of the intracellular NO level

Yeast cells cultured until early exponential phase in SD medium were treated with 10 μM DAF-FM DA for 30 min, followed by addition of 0.5 mM NOC-5 or a vehicle 0.17 mM NaOH. After incubation at 30 °C for the indicated time, cells were diluted in 10-fold and then the intracellular fluorescence intensity was monitored by flow cytometry (FCM) using BD Accuri C6 Flow Cytometer (Becton, Dickinson Bioscience). The relative fluorescence intensity was calculated as described previously^20^.

### Western blot analysis

After culture or treatment, harvested yeast cell were treated with 0.3 M NaOH for 10 min, pelleted, resuspended in SDS-sample buffer containing 4% SDS and 4% 2-mercaptoethanol, and then boiled for 3 min to extract proteins^37^. The extracted proteins were separated by SDS-polyacrylamide gel electrophoresis (SDS-PAGE), followed by immunoblotting. The visualization of immunoblot was performed using Pierce™ ECL Plus Western Blotting Substrate (Thermo Fisher Scientific) and LAS-4000 imager (Fujifilm).

### Preparation and enzymatic activity assay of the recombinant Rib1p

One hundred μL of overnight culture of *E. coli* Rosetta (DE3) cells harboring pET53-RIB1 in LB medium containing chloramphenicol and ampicillin was inoculated into 100 mL of the fresh same medium, and further cultivated for 6–7 h at 30 °C. Subsequently, the culture was incubated at 18 °C for 30 min, followed by addition of 1 mM IPTG and 1% glucose. After additional incubation for 16–20 h at 18 °C, the cells were pelleted, washed with, and suspended in lysis buffer containing 50 mM Tris-HCl (pH 8.0), 0.5 M NaCl. Next, cell suspensions were subjected to sonication and centrifugation to prepare cell lysate. Extracted lysate was mixed with Ni-NTA agarose, and then rRib1p was purified through wash and elution using lysis buffer with 50 mM and 500 mM imidazole, respectively. After buffer exchange to 50 mM Tris-HCl (pH 8.0), 1 mM TCEP, 10% glycerol using Amicon^®^ Ultra 10 K (Merck), the sample containing rRib1p was used further analyses. SDS-PAGE and CBB staining were performed to observe the protein purity. The GTPCH2 activity of the purified Rib1 was measured by monitoring the fluorescence derived from 6,7-dimethylpterin, which is a reaction product of DARP and diacetyl, using a spectrofluorometer F-7000 (Hitachi), as previously described^38,39^. To quantify generated DARP, the standard curve was drawn using various concentrations (0–50 μM) of 6,7-dimethylpterin. One unit of the specific activity of GTPCH2 was defined as the amount of enzyme to produce 1 μmol of DARP per min.

### Preparation of the DARP-containing solution

The GTPCH2 reaction of rRib1p was performed in the solution containing 100 mM Tris-HCl (pH 7.4), 5 mM MgCl_2_, 5 mM DTT, 50 μM GTP, and rRib1p by incubation at 37 °C for 2 h. Enzyme was inactivated by boiling for 5 min and the supernatant after centrifugation was used for further analyses as a DARP containing solution. The concentration of DARP contained in the sample was measured using diacetyl and a spectrofluorometer F-7000 (Hitachi) as described above and previously^38,39^.

### *In vitro* NO quenching assay

To examine the NO degrading activity of rRib1p, rRib1p was incubated in the solution containing 100 mM Tris-HCl (pH 7.4), 5 mM MgCl_2_, 5 mM DTT, 7 μM DAF-FM, 100 μM NOC-5 without GTP, and subsequently the fluorescence intensity was monitored over time using a spectrofluorometer F-7000 (Hitachi) with 500 nm of excitation and 515 nm of emission wavelength. To examine the NO scavenging activity of DARP or TAPO, a DARP containing solution prepared as above or a various concentrations of TAPO was incubated in the solution same as above without rRib1p. To evaluate the NO quenching effect of other compounds related with the GTPCH2 reaction, 100 μM pyrophosphate, 100 μM GTP, or 100 μM formate was added instead of DARP or TAPO. The fluorescence intensity was measured as above.

## Supplementary information

Supplementary Information.
