# Supplementary material for: A Novel Mechanism for Nitrosative Stress Tolerance Dependent on GTP Cyclohydrolase II Activity Involved in Riboflavin Synthesis of Yeast
Source: Sci Rep. 2020 Apr 7;10:6015. doi: 10.1038/s41598-020-62890-3 (PMC7138843; doi:10.1038/s41598-020-62890-3)
Supplement: Supplementary file 1 — Supplementary Information. [file 41598_2020_62890_MOESM1_ESM.docx]

**Supplementary Information**

A Novel Mechanism for Nitrosative Stress Tolerance Dependent on GTP Cyclohydrolase II Activity Involved in Riboflavin Synthesis of Yeast

Khairul Anam^1,2ǂ^, Ryo Nasuno^1ǂ^, Hiroshi Takagi^1^*

ǂ These authors equally contribute to this manuscript.

^1^Division of Biological Science, Graduate School of Science and Technology, Nara Institute of Science and Technology, 8916-5, Takayama-cho, Ikoma, Nara 630-0192, Japan

^2^Research Center for Biotechnology, Indonesian Institute of Sciences, Jl. Raya Bogor KM 46, Cibinong 16911, Bogor, West Java, Indonesia

*Correspondence e-mail: [hiro@bs.naist.jp](mailto:hiro@bs.naist.jp)

**Contents**

**Supplementary Figures S1-S3**

**Supplementary Table S1**

**Supplementary Figure 1**


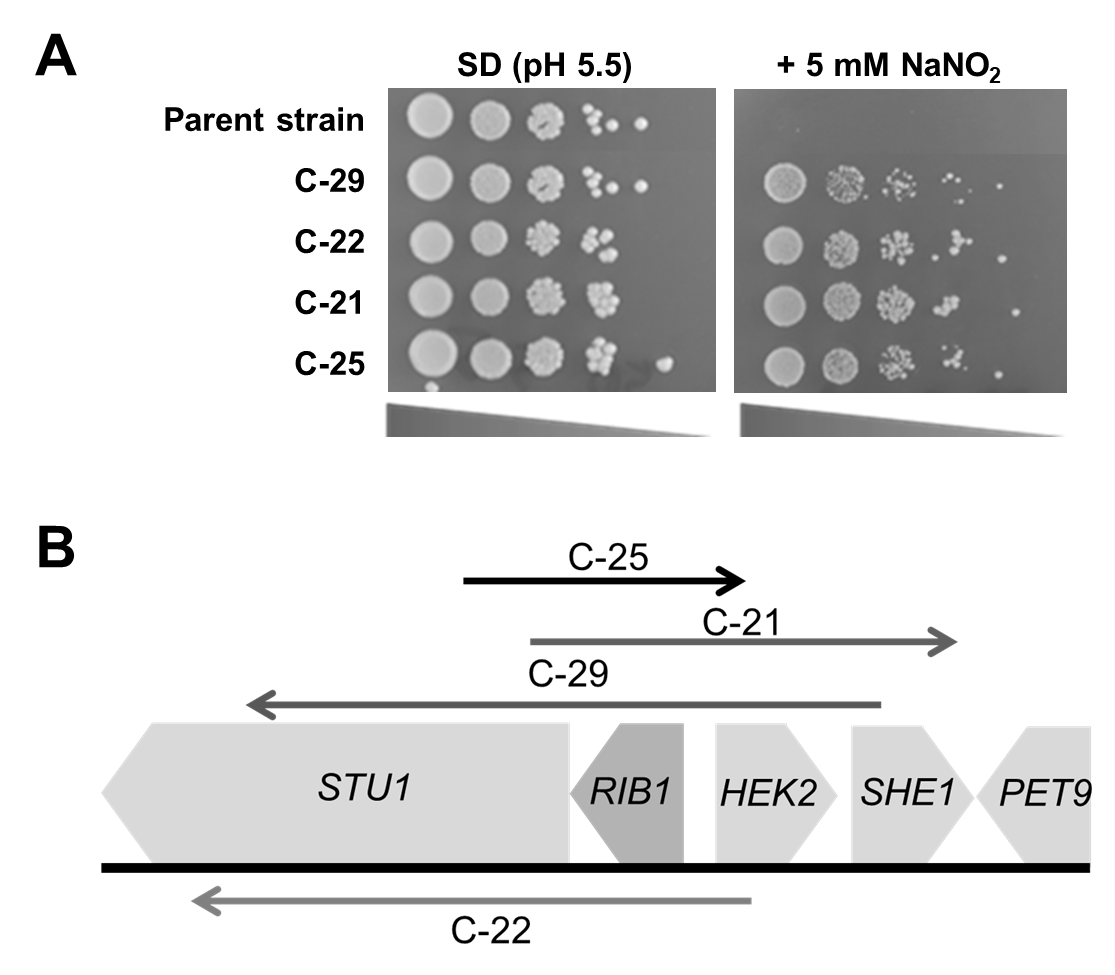


**Figure S1. Screening and identification of the *RIB1* gene as a novel NO tolerance gene.** (A) Spot assay for NO tolerance or candidate clones isolated from the first screening. Yeast cells grown until early exponential growth phase were serially diluted and spotted onto SD medium with pH 5.5 containing 5 mM NaNO_2_. (B) DNA regions harbored in the isolated library plasmids extracted each candidate clone. The description C-21, 22, 25, or 29 corresponds to the clones shown in panel A.

**Supplementary Figure 2**

**
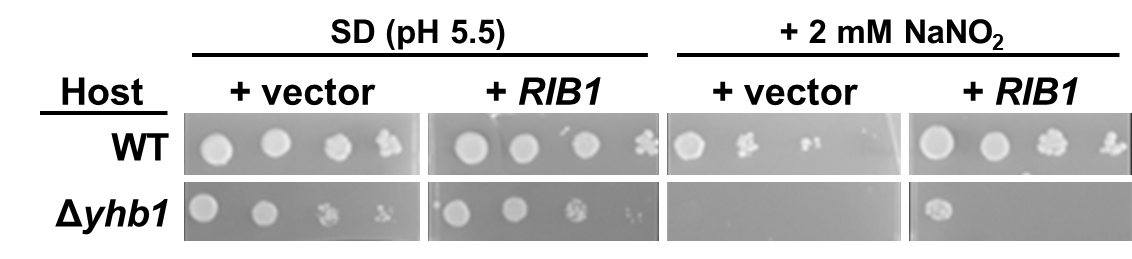
**

**Figure S2. Effect of *YHB1* on RIB1-dependent nitrosative stress tolerance.** WT or Δ*yhb1* cells harboring empty vector or overexpressing *RIB1* cultured until exponential growth phase were serially diluted and then spotted onto the acidified medium with NaNO_2_


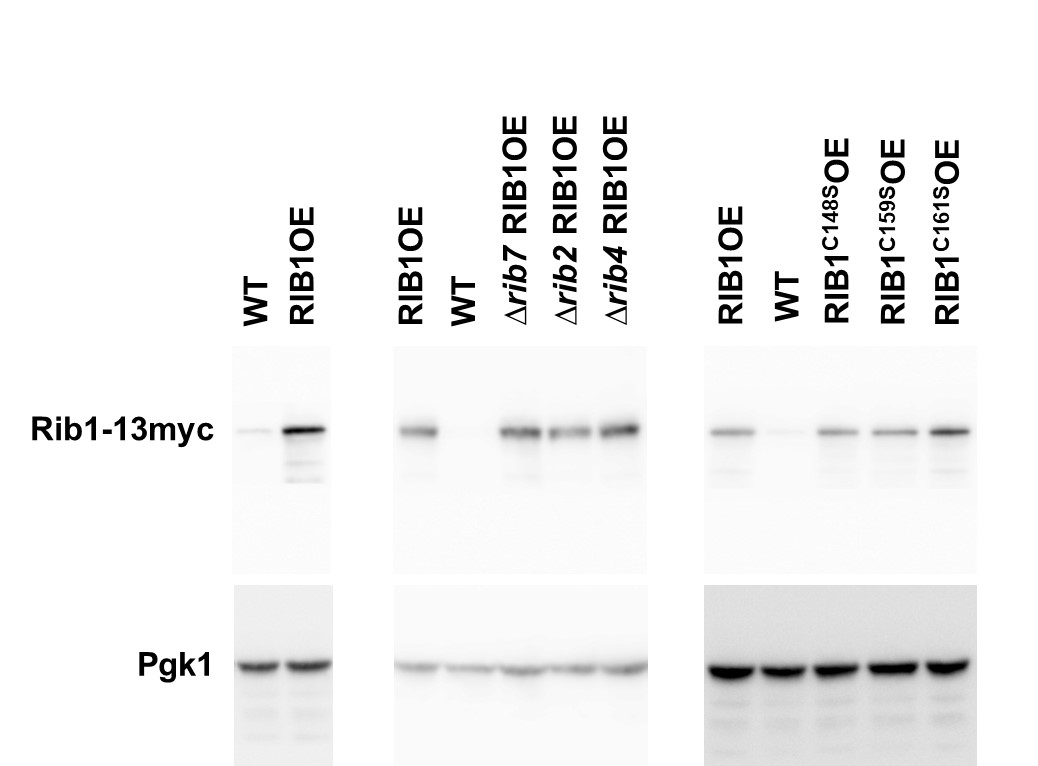
**Supplementary Figure 3**

**Figure S3. Western blotting to detect Rib1.** The pictures of Western blotting for Rib1 detection in untrimmed membranes were shown. Left, middle, and right panels correspond to Fig. 1B, Fig. 3C, and Fig. 4C, respectively.

**Table S1. Oligonucleotide primers used in this study**

| Primers | DNA sequence (5’-3’) | Description |
| --- | --- | --- |
| S1_RIB1_Fw | acaaaaagtggcgtacataaaactacaacaaacctacaggatgCGTACGCTGCAGGTCGAC | *RIB1* disruption |
| S2_RIB1_Rv | aaaagcactatatgtactaataattatgctacacttgtgtttaATCGATGAATTCGAGCTCG | *RIB1* disruption |
| S3_RIB1_Fw_new | aactctgcgttgtcatcaacatcgacgctggcaataTCGTACGCTGCAGGTCGA | *RIB1* tagging on genome |
| S4_RIB1_Rv | tatttgctgctatcctgtttactgttgtcgtagttatctatggtCATCGATGAATTCTCTGTCG | *RIB1* tagging on genome |
| S1_RIB2_Fw | aagaacattaaaaaaaaaactatcaatattagattaacaaaccaatcaaaatgCGTACGCTGCAGGTCGAC | *RIB2* disruption |
| S2_RIB2_Rv | gtggttattgtagatatgatgtaagattacatgataatatatatgctaaatagtttaATCGATGAATTCGAGCTCG | *RIB2* disruption |
| S1_RIB4_Fw | aaggaacagtataacgcagtataacgcagtataacgcagtataacgcagtatgCGTACGCTGCAGGTCGAC | *RIB4* disruption |
| S2_RIB4_Rv | ttctatagtgagtatatatacatatatagtatagatttctctgcgcttatttaATCGATGAATTCGAGCTCG | *RIB4* disruption |
| S1_RIB7_Fw | accaacctattggtagcttctttgtattactatcaacatttttagaagatatgCGTACGCTGCAGGTCGAC | *RIB7* disruption |
| S2_RIB7_Rv | ctaatttcagaaagaaattacacagaaggtggagttctaacactattaATCGATGAATTCGAGCTCG | *RIB7* disruption |
| RIB1_Gateway_Fw | ggggacaagtttgtacaaaaaagcaggcttaATGACCATAGATAACTACGA | Plasmid construction |
| 13myc_Gateway_Rv | ggggaccactttgtacaagaaagctgggtgCTAGTGATTGATTAATTT | Plasmid construction |
| C148S_Fw | AGTTACACCGGTGAAAACGC | Point mutation |
| C148S_Rv | TTCAGAATGGATCCGTACCAGCG | Point mutation |
| C159S_Fw | AGTGATTGTGGTGAACAATTCG | Point mutation |
| C159S_Rv | ACGGGCGCTCCATGCGTTTTC | Point mutation |
| C161S_Fw | AGTGGTGAACAATTCGATAGGG | Point mutation |
| C161S_Rv | ATCACAACGGGCGCTCCATG | Point mutation |

The capital case letters indicate the primer sequence which attaches to the template DNA during PCR. The mismatched sequences to introduce point mutations are underlined.
